# Supplementary figures and images for: The predictive value of universal preschool developmental assessment in identifying children with later educational difficulties: A systematic review
Source: PLoS One. 2021 Mar 4;16(3):e0247299. doi: 10.1371/journal.pone.0247299 (PMC7932552; doi:10.1371/journal.pone.0247299)

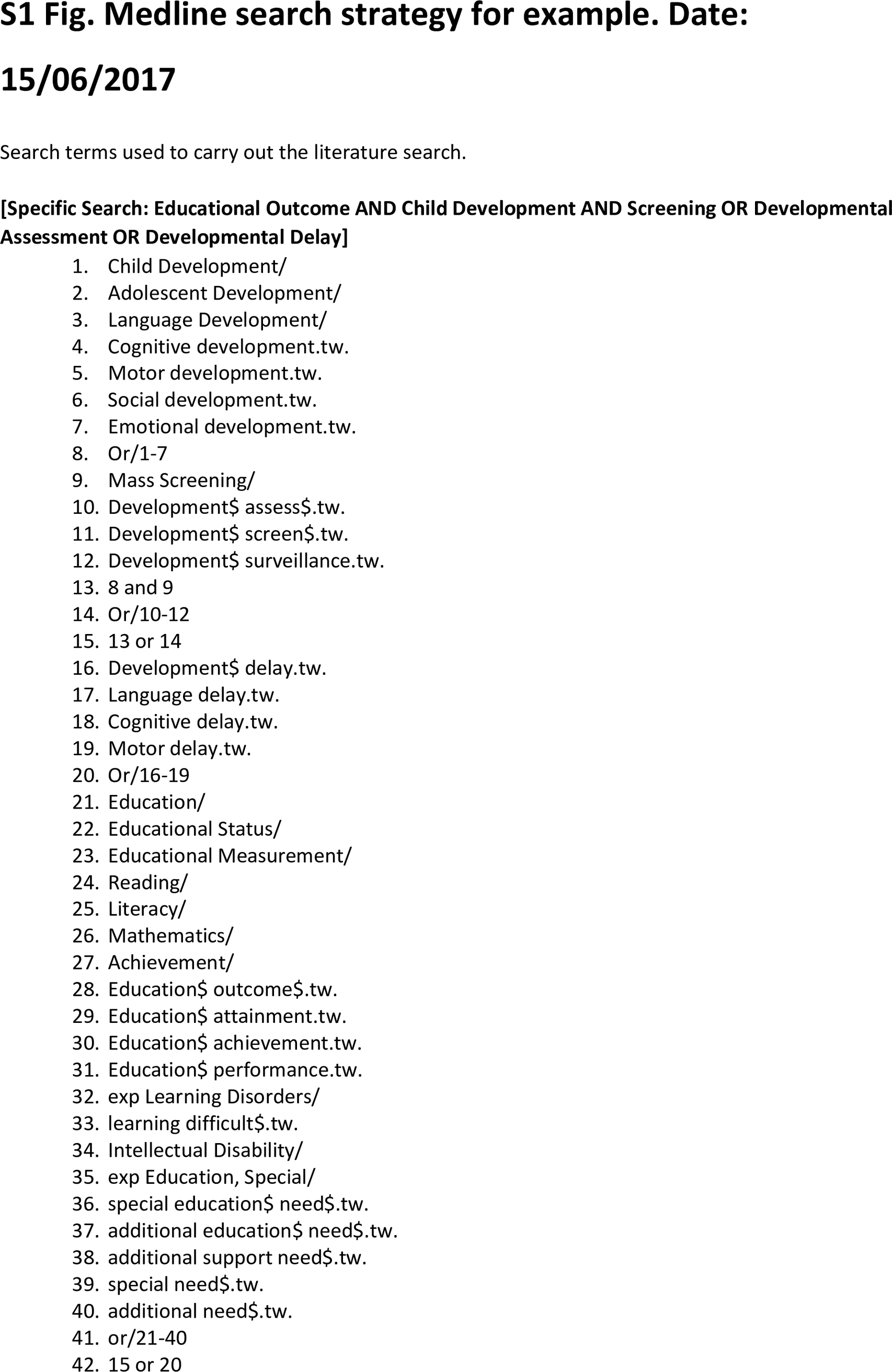

Supplement: S1 Fig — Medline search strategy for example. Date: 15/06/2017. (TIF) [file pone.0247299.s001.tif]

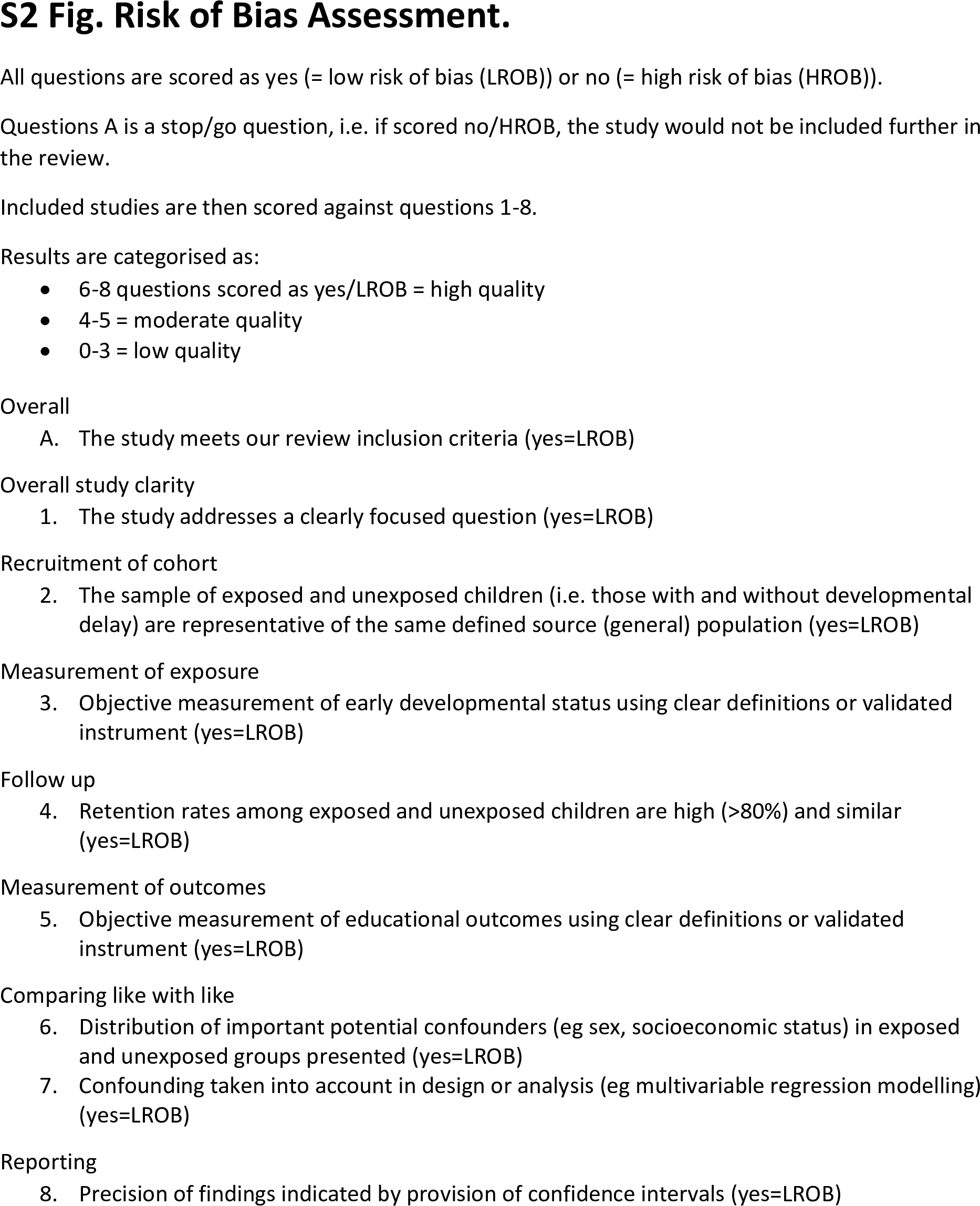

Supplement: S2 Fig — All questions are scored as yes (= low risk of bias (LROB)) or no (= high risk of bias (HROB)). Questions A is a stop/go question, i.e. if scored no/HROB, the study would not be included further in the review. Included studies are then scored against questions 1–8. Results are categorised as: 6–8 questions scored as yes/LROB = high quality4–5 = moderate quality0–3 = low quality. (TIF) [file pone.0247299.s002.tif]

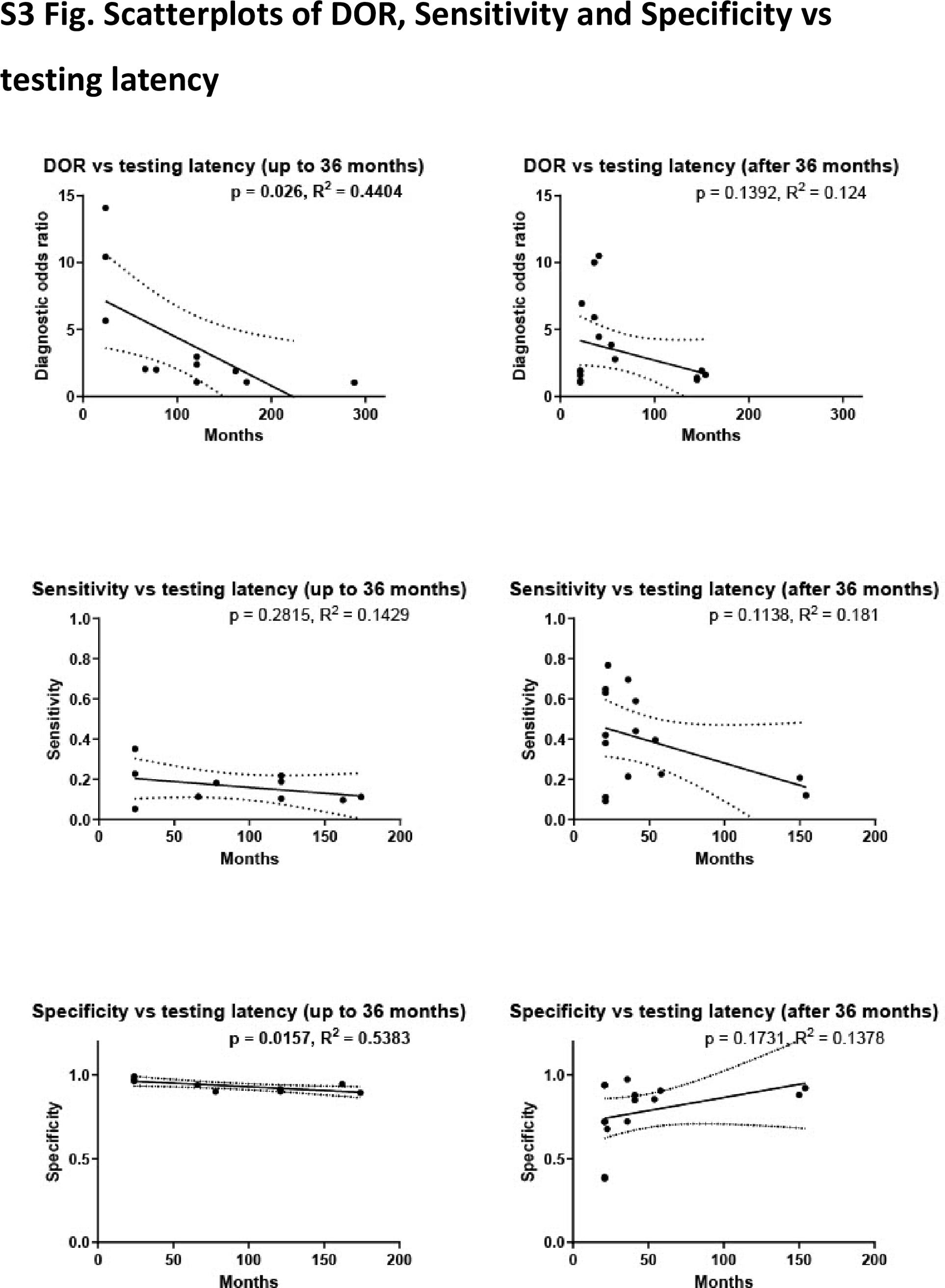

Supplement: S3 Fig — Scatter plots of diagnostic odds ratios, sensitivity and specificity versus latency between initial assessment and school assessment in months: Results shown separately for studies with initial developmental assessment conducted prior to 36 months (left) and at 36 months or later (right). Linear regression is demonstrated with p values (results < 0.05 in bold) and R2 values for goodness-of-fit indicated. (TIF) [file pone.0247299.s003.tif]
