# Supplementary material for: The predictive value of universal preschool developmental assessment in identifying children with later educational difficulties: A systematic review
Source: PLoS One. 2021 Mar 4;16(3):e0247299. doi: 10.1371/journal.pone.0247299 (PMC7932552; doi:10.1371/journal.pone.0247299)
Supplement: S1 Table — Template used to extract data for studies included in the review. (PDF) [file pone.0247299.s004.pdf]

S1 Table. Bespoke data extraction template for included studies

Template used to extract data for studies included in the review.

Study identification

First author surname	
Article title	
Journal	Official abbreviated journal name<br>https://wilkes.libguides.com/c.php?g=191948&p=1266554
Citation	Year; Volume(Issue): Start page-end page
doi	e.g. doi: 10.1001/jama.2010.1275
Covidence id number	#
Corresponding author institution	e.g. University of Edinburgh
Funding source	Note if funding source not stated
Risk of bias score	0-8
Study quality category	Categorised as<br>• High (6-8)<br>• Moderate (4-5)<br>• Low (0-3)

Study design

Study design category	Categorised as<br>• Intervention study<br>• Cohort study<br>• Case control study<br>• Other
Cohort study category	Categorised as<br>• Follow up of children receiving an established child health programme<br>• Follow up of children in population based birth cohort study<br>• Other<br>• Not applicable<br>Note that for either type of cohort study, outcomes could be assessed through direct follow up/primary data collection or through linkage to routine education records.<br>Although cohort studies are conceptually prospective, studies may have been done retrospectively ie starting with educational outcomes and linking back to prior developmental information.

Population

Setting	Country
Age of children at initial developmental assessment	In months
Year of initial developmental assessment	Calendar year(s)
Inclusion criteria - detail	e.g. children resident in Lothian attending the universal 27 month child health review between 2010 and 2015 inclusive OR<br>children in the UK Millennium Cohort Study who underwent developmental assessment at the age 3 data collection in 2003
Exclusion criteria	e.g. children with diagnosed neurodevelopmental condition or intellectual disability
Number of children eligible for inclusion	May be complex eg for study based on birth cohort could capture<br>• Number invited to participate in cohort<br>• Number participating in first sweep<br>• Number participating in sweep involving baseline developmental assessment<br>For study based on routine care in a specified area could capture<br>• Resident population of the age undergoing baseline developmental assessment (prospective cohort)<br>• In school population of the age undergoing educational outcome assessment (retrospective cohort)<br>Record if size of target population not stated in paper

Intervention (developmental assessment)

Developmental assessment - category	Categorised as<br>• Parental concerns<br>• Clinical assessment (developmental history, examination, observation)<br>• Validated assessment tool (parental questionnaire such as ASQ, professionally administered questionnaire such as Denver II, professionally administered developmental test such as BAS)<br>• Other
Developmental assessment – domain	Domain(s) of development assessed, categorised as<br>• Cognitive and problem solving<br>• Speech, language and communication<br>• Motor<br>• Social and emotional<br>• Other
Developmental assessment – detail	e.g. overall Health Visitor assessment of child's development by domain based on elicitation of parental concerns, developmental history, structured observation, and parental completion of ASQ as described in national guidance for Scottish child health programme http://www.gov.scot/Publications/2012/12/1478/0 OR administration of the naming vocabulary assessment and picture similarities subtests of the British Ability Scales II as part of the Millennium Cohort Study age 3 data collection

Comparator (children with and without developmental delay/concern)

Definition of developmental delay/concern	e.g. Health Visitor identified a new or existing concern about the child's development (any domain) at the end of the 27 month child health review OR<br>child scored >1SD below mean for age and gender based on standard UK norms for BAS II (either subtest)
Children with developmental delay/concern – number	Study may report an overall exposed group or by specific subgroups e.g. children with difficulties in specific developmental domains
Children with developmental delay/concern – characteristics	Baseline characteristics likely to be important confounders, such as<br>• Mean age at assessment<br>• % male<br>• % lowest socio-economic group<br>• Other
Children without developmental delay/concern – number	As for exposed group(s)
Children without developmental delay/concern – characteristics	As for exposed group(s)

Outcomes (educational outcomes and results)

Educational outcome(s) – category	Categorised as<br>• Educational attainment – general<br>• Educational attainment – subject specific<br>• Recognition of additional educational needs/special schooling<br>• Academic ability/IQ<br>• Attendance or exclusion<br>• Other
Educational outcome(s) – detail	e.g. additional educational needs as recorded on the annual pupil census according to national guidance http://www.gov.scot/Topics/Statistics/ScotXed/SchoolEducation/SchoolPupilCensus/SurveyDocumentation OR administration of the WIAT-II as part of the Millennium Cohort Study age 8 data collection
Definition of adverse educational outcome(s)	e.g. additional education need recorded on annual pupil census (any underlying reason) OR<br>child scored >1SD below mean for age and gender based on standard UK norms for WIAT-II
Age of children at assessment of educational outcomes	In years
Year of assessment of educational outcomes	Calendar year(s)
Children with developmental delay/concern with outcome information – number	
Children with developmental delay/concern – results	Raw results eg number and % OR mean and SD of exposed children with adverse educational outcome
Children without developmental delay/concern with outcome information – number	
Children without developmental delay/concern – results	As for exposed group(s)
Measure of association between early developmental delay/concern and later adverse educational outcomes - category	Categorise as<br>• Relative risk<br>• Risk difference<br>• Other
Measure of association between early developmental delay/concern and later adverse educational outcomes – raw results	Include point estimate and CI
Measure of association between early developmental delay/concern and later adverse educational outcomes – adjusted results	Include point estimate and CI
Concern about selective reporting	Any evidence that only educational outcomes showing positive association with earlier developmental delay/concerns reported?
