# Supplementary material for: The predictive value of universal preschool developmental assessment in identifying children with later educational difficulties: A systematic review
Source: PLoS One. 2021 Mar 4;16(3):e0247299. doi: 10.1371/journal.pone.0247299 (PMC7932552; doi:10.1371/journal.pone.0247299)
Supplement: S2 Table — (PDF) [file pone.0247299.s005.pdf]

S2 Table. Percentage of at risk individuals on preschool assessment and incidence of adverse educational outcomes

Table 4: Percentage of at risk individuals on preschool assessment and incidence of adverse educational outcomes

	**Initial developmental screening**			**Educational outcome assessment**		
Study [Reference no.]	Age at initial test (months)	Developmental screening methods	Percentage at risk on initial assessment	Age at follow-up (years)	Educational outcome methods	Incidence of adverse educational outcome
Bleses *et al* [24]	16-30 months	Danish Communicative Development Inventories (CDI) lowest 10%	9.9%	12 years	Lowest 10% for language comprehension	10.0%
					Lowest 10% language decoding	10.0%
					Lowest 10% reading comprehension	10.0%
Cadman *et al* [25]	48-60 months	Model 2: Abnormal DDST; health, development and behavioural history	20.7%	8 years	Composite measure of performance at the end of the third school year	18.4%
		Model 4: Abnormal DDST; health, development and behavioural history; and kindergarten teacher rating	20.4%			18.4%
Charkaluk *et al* [16]	37.3 ± 0.8 months	ASQ score < 270	35.6%	5 years	IQ < 85 on WPPSI-III	7.3%
Murray *et al* [19]	24 months	Age of saying words other than names of parents	Not available	26 years	Progression to "A" levels	Not available
Paget *et al* [20]	18 months	Lowest 10% for general development	10.0%	8 years	Exclusion by 8 years	0.6%
			10.7%	16 years	Exclusion by 16 years	8.4%
	30 months	Maternal concerns re behaviour development	5.9%	8 years		0.6%
			5.6%	16 years		8.0%
	38 months	Lowest 10% for language (MacArthur Questionnaire)	9.5%	8 years		0.6%
			8.1%	16 years		8.0%
	42 months	SDQ (≥14)	14.6%	8 years		0.6%
			12.6%	16 years		8.2%
Silva [21]	36 months	Motor issue	1.4%	5 years	Composite measure - IQ, co-ordination, verbal skills	10.4%
		Language issue	4.8%			10.4%
		Motor +/- language issue	6.9%			10.4%
		Motor +/- language issue	6.9%		Stanford Binet IQ < 77	2.7%
Smithers *et al* [22]	48-60 months	Parent reported language/ear/hearing problems	29.5%	6-7 years	<1 SD below the mean on maths ARS	15.0%
			29.8%		<1 SD below the mean language/literacy ARS	13.2%
		Parent reported gross/fine motor difficulties	6.4%		Maths	15.0%
			6.7%		Language/literacy	13.2%
		Parents reported issues with sleep, emotional wellbeing, energy/activity levels or being nervous/clingy	61.6%		Maths	15.0%
			62.1%		Language/literacy	13.2%
Valtonen et al [27]	48 months	Lene4 test (0.16 least stringent cut off)	36.2%	7 years	JLD Teacher Questionnaire - lowest 20% academic section	19.9%
		Lene4 test (0.50 most stringent cut off)	6.4%			19.8%
Washbrook *et al* [23]	47 months	Hyperactivity/inattention problems on SDQ	Not available	16 years	Failure to achieve five A*–C GCSEs (male)	Not available
					Failure to achieve five A*–C GCSEs (female)	
		Conduct problems on SDQ			Failure to achieve five A*–C GCSEs (male)	
					Failure to achieve five A*–C GCSEs (female)	
