# Supplementary material for: The predictive value of universal preschool developmental assessment in identifying children with later educational difficulties: A systematic review
Source: PLoS One. 2021 Mar 4;16(3):e0247299. doi: 10.1371/journal.pone.0247299 (PMC7932552; doi:10.1371/journal.pone.0247299)
Supplement: Attachment — Submitted filename: Reponse to reviewers_4Dec20_FINAL SUBMITTED.docx [file pone.0247299.s006.docx]

**Journal Requirements:**

1. Please ensure that your manuscript meets PLOS ONE's style requirements, including those for file naming. The PLOS ONE style templates can be found at

<https://journals.plos.org/plosone/s/file?id=wjVg/PLOSOne_formatting_sample_main_body.pdf> and

<https://journals.plos.org/plosone/s/file?id=ba62/PLOSOne_formatting_sample_title_authors_affiliations.pdf>

*This has been done.*

 2.In your Methods section, please specify the date of your search.

*This has been added in S1 Fig.*

 3.Thank you for stating the following financial disclosure:

 [This research was funded from a charitable donation to the Salvesen Mindroom Research Centre. The funder had no role in the design, conduct, or reporting of the review.].

 We note that one or more of the authors is affiliated with the funding organization, indicating the funder may have had some role in the design, data collection, analysis or preparation of your manuscript for publication; in other words, the funder played an indirect role through the participation of the co-authors. If the funding organization did not play a role in the study design, data collection and analysis, decision to publish, or preparation of the manuscript and only provided financial support in the form of authors' salaries and/or research materials, please do the following:

1. Review your statements relating to the author contributions, and ensure you have specifically and accurately indicated the role(s) that these authors had in your study. These amendments should be made in the online form.
2. Confirm in your cover letter that you agree with the following statement, and we will change the online submission form on your behalf:

“The funder provided support in the form of salaries for authors [insert relevant initials], but did not have any additional role in the study design, data collection and analysis, decision to publish, or preparation of the manuscript. The specific roles of these authors are articulated in the ‘author contributions’ section.”

*This has been done.*

3. Please include captions for your Supporting Information files at the end of your manuscript, and update any in-text citations to match accordingly. Please see our Supporting Information guidelines for more information: <http://journals.plos.org/plosone/s/supporting-information>.

*This has been done.*

**Additional Editor Comments (if provided):
Major comments**

The apparent contrast between the results of Sim et al. (2019) and the present study is worth further discussion. To what extent are the findings reconcilable? For example, it seems possible that predicting language and behavioral difficulties might naturally require a different screening/assessment approach than predicting educational outcomes. In addition, the screening tools that were analyzed in the present study vary in the extent to which they load on language. If public health programs aim to develop research-based screening protocols, they will likely be interested in identifying the smallest set of instruments that predict the widest range of outcomes. Considering the results of Sim et al. (2019) alongside those of the present study, what recommendations or cautions are in order?

*We have added in a paragraph about the differences in both predictors and outcomes explored in the two studies and how these may be fundamentally different (pg24):*

*This may be reflective of the different measures being examined: Sim et al. looked specifically at language and/or behaviour as predictors of later development, compared with our focus on developmental delay, which may or may not encompass language and/or behaviour among other elements. Furthermore, the outcomes examined differed, with Sim et al., looking for associations with developmental delay around the start of school, compared with the current study which explored predictors of later educational difficulties. It may be that these are fundamentally different, with factors associated with later educational delay being broader than those associated with relatively early developmental delay.*

I would like to see a more thorough discussion of the potential dissociability of age at initial screening, age at outcome measurement, and elapsed time. It is unsurprising that less elapsed time results in greater predictive validity. However, it is presently unclear whether the lower predictive validity of screening at younger ages is actually reflective of developmental phenomena, as the manuscript currently suggests, or whether this is a confound of earlier screenings being used to make predictions across longer timespans. I would encourage the authors to either address this point more directly, or to explicitly state what prevents this question from being addressed. Again, those responsible for designing screening programs will surely want to know whether to, for example, attempt to screen once early on, once later on, or twice (where the earlier screen predicts on a much shorter timespan). It seems to me that the present data may be able to speak to that question; I encourage the authors to comment on it.

*Thank you – this was an interesting suggestion. We have added some further discussion around this point. In addition, we have produced a set of supplemental tables which divide the data up to initial assessments before and after 36 month, and then results are interesting and seem to support our feeling that latency is more important at younger ages where developmental trajectories are more unstable, although coupled with the children in the older studies tending to have relatively shorter follow-up periods. Pg16/S3 Fig. We have also added in a further sentence around this to the discussion – pg25.*

I would like the authors to be more explicit about their vision for the practical application of the knowledge gained from this study. For instance, I initially wondered whether the goal was to understand what high-income countries are doing so that low-income countries might learn from their example, but this was (in my view, rightly) recognized as a limitation later on. Is the goal to make recommendations for high-income countries who are not yet doing early surveillance? To help countries that are doing population-based screening do so more efficiently? Something else?
I write from an American perspective, where there is considerable concern that many of our standardized outcome measures unjustly reflect prejudices regarding differences in race, ethnicity, and class, among others. Thus, some of the variability in predictive accuracy is attributable to measurement error (in screening and/or eventual outcomes). I invite the authors to comment on the extent to which this may be a problem in the extant research. For example, to what extent to the populations tested resemble the norming samples of the measures used? To what extent have the measures themselves been evaluated to determine whether they inappropriately penalize or pathologize diversity?

*This is a good point. We have added the lack of examination of tools reflecting bias into the limitations. We have also added a more explicit explanation of our vision at the start of the discussion (Pg25).*

**Additional comments**

Introduction: The manuscript makes a strong case for the expected impact of early population-based surveillance/assessment, but no information is provided about the ultimate impact of these efforts. Including this type of evidence would help non-specialists to understand the value of the present study and its potential long-term impact.

*We have added in further information on the evidence around the impact of early screening (page 4, lines 69-78)*

Table 1: What was the rationale for excluding diagnostic studies, if they met the other inclusion criteria?
*Our review focuses on population based studies conducting initial surveillance of early child development as understanding the predictive validity of this public health activity was the main aim of our study. Within this, we included studies providing information on the specified educational outcomes of interest. We excluded studies that examined the predictive ability of initial developmental surveillance in relation to diagnosis of specific developmental disorders (unless they also provided information on included educational outcomes) as that was not our focus of interest.*

Information Sources: In the introduction, the authors (justifiably) critique a previous review for having been conducted 20 years ago. What then is the rationale for including studies from the mid-20th century, especially given the substantial changes to our understanding of child development, disorders, and diagnostics since that time?

*The issue with that systematic review was that it contained no new studies from the last 20 years, due to when it was conducted. Our database search focused on papers published from 2000 onwards (this is clear from our search strategy in the Appendix). Earlier papers were therefore only included if identified through searching of ref lists, the output lists of major cohort studies, or recommendation from our expert advisory group, and hence likely to be of particular importance to the evolving evidence base.*

Data Extraction: Data on the rates of initial agreement between the coders would be appreciated, either as supplemental material, or in summarized form in the main text.

*Agreement at the title and abstract level was fair (Cohen's Kappa 0.31) (Landis and Koch, 1977). At the full text review level agreement was good to very good (Cohen's Kappa 0.55 - 1) depending on which reviewers were compared as multiple reviewers were involved at this level. Agreement was good for the risk of bias assessment (Cohen's Kappa 0.62).*

*At the data extraction level, two reviewers independently extracted data and disagreements were discussed however due to the relatively fluid discussions about which data to extract from very heterogeneous papers it was not possible to quantify this. We will keep this in mind on future reviews.*

*Landis, J.R.; Koch, G.G. (1977). The measurement of observer agreement for categorical data. Biometrics. 33 (1): 159-174. https://doi.org/10.2307%2F2529310*Typographical comments:

There are a number of small syntactic or word-level errors throughout the manuscript (e.g. ln. 74-76; ln 290, inter al.). Please proofread carefully in preparation for the next submission.
Similarly, there are several instances where a study is named without an accompanying citation number.

*We have proof read and hope we have spotted everything this time.*


Reviewers' comments:
**Reviewer #1:**

The manuscript is well written and presents data on a question that is important for the field of communication disorders and sciences. These findings are important as we move forward and continue to develop various child-find and early intervention screening measures. The comments and suggestions made below are meant to add to the rigor of the paper, ensure that these findings can be replicated, and that the methods can be used in similar studies. All of these comments are justifications for my decision in question one.

The authors should describe what are "acceptable" or "good" levels of sensitivity and specificity. I would suggest that the authors done by Plante and Dollaghan.

*This has been added in the Summary measures section (line 185-186)*

The the method section specifically lines 118-121 you should give your inclusionary criteria for your initial search.

*We believe we have provided a full account of this information in the ‘Eligibility Criteria’ section of the methods, as well as in Table 1 and S1 Fig, and think it may not be required to repeat this earlier.*

The authors should describe what is the criteria to be considered a "high income country"? Authors should provide a rationale for only including high income countries.

*I have added a few sentences into the methods about the criteria and justification used for HICs (pg7).*

The authors discuss doing a special search just for articles that used the Ages and Stages Questionnaire. Why is this? Is there potential bias for the results for doing an additional search for one screening tool and not the others? This needs to be grounded in a theoretical framework and should be considered as a limitation is this is a potential source for bias.

*The rationale was that this is the current screening tool in Scotland and England and we were therefore keen to explore the results around this specific measure. The additional search only yielded one study (Charkaluk 2017) and this had already been identified in the database search. It could have been a source of bias if further studies were discovered, however we believe that it just confirmed a robust database search strategy.*

A rationale and framework for why the specific search terms were selected is needed so that the rigor can be evaluated and methods and results can be replicated.

*We have expanded on this in the text – pg8.*

Lines 202-204: What is meany by "inconsistent and inadequate reporting"? This is important to fully evaluate the methods used and to make sure important article findings were not discarded.

*This means that different studies reported different measures. We have added a clarification into this sentence.*

Line 209: Why are the combination of screening tools "cumbersome to administer"? This needs to be more clear and should be given more attention in the discussion as this is of critical importance for implementation.

*We have changed ‘cumbersome’ to ‘resource intensive’ to hopefully make clearer what we mean.*

The results need to be more organized and more clear. The authors seem to jump back and forth between the screening tools and their diagnostic ability/accuracy. I would suggest given more general results and then give specific results in section for each screening tool you are discussing to make sure that this is easier to read for the audience.

*We discussed the presentation of results at length, and there of course pros and cons as to different ways of presenting them. We have regrouped the results on studies with DORs to reflect the groupings in Figure 3.1, with the broad overview at the top of this section, which we hope is clearer. We have left the studies with other association measures as they are as these were felt to be too heterogenous to group.*

**Reviewer #2:**

This article addresses an important question, and the systematic review is well conducted, although it does not have a clear conclusion due to the heterogeneity of the studies included.

I have some comments:

- The research is about “universal preschool developmental assessment”. I understood it refers to the same framework as “screening of developmental difficulties in the population”. In this context, predictive value of baby-tests is not studied. The authors should more clearly set this point from the beginning of the article.

*We think that by ‘baby tests’ the reviewer is referring to the newborn physical examination that is offered as an initial screen in many settings (including Scotland). This is more of a general physical examination, for example looking for congenital anomalies such as heart defects, rather than a developmental assessment per se, although obviously there is some overlap between the two. The newborn exam will usually include an assessment of hearing and vision, but note we excluded studies if the initial assessment only focused on/reported sensory development. It will also usually include an assessment of gross motor function. The newborn exam would also be expected to detect complex anomalies/syndromes likely to affect babies’ cognitive development, for example Down syndrome. We did not identify any population based studies examining the association between developmental problems detected in the immediate neonatal period and children’s later educational outcomes. Although no screening tests should be accepted as a self-evidently ‘good thing’, this is perhaps not surprising as it is likely that developmental problems detected at such an early stage reflect serious underlying conditions that can be expected to have a significant ongoing impact on children. The studies we identified involved developmental assessment at older (but still pre-school) ages, when more subtle developmental problems emerge and can be identified. The predictive significance of these later developmental ‘delays’ (and hence also the potential value of a population based surveillance or screening approach to identifying them) is much more strongly contested and hence the focus of more active research.*

- About full inclusion and exclusion criteria (table 1), age range is specified for the population, ie 0-60 months. But no age range is specified for outcomes. In the text, it is quite surprising to see that there is an overlap between age range for preschool assessments and later educational difficulties, which begins at 4 years in ref [24].

*No age range was defined due to the wide potential definition of education and the times at which this is assessed. For instance Murray et al 2007 assessed O-level vs A-level attainment at age 26 years. Although these qualifications are usually obtained at 16 and 18 years respectively, there may be some variation in this and some individuals may achieve qualifications at this level beyond 18 years (such as in a non-university college setting).*

*With regards to the overlap in age ranges for preschool assessments and later educational difficulties, the paper in question (Duff et al., 2015) notes that there was only 1 child of 4 years of age (who technically was in school in that UK population) and thus it was considered acceptable to include this paper, the division being between preschool assessment and later educational outcomes.*

- In the same field, there is a latency of only 6 to 12 months in ref [22]. Is this study really in the topic of the current paper?

*Ref 22 (Fowler et al, 1986): Our understanding is that minimum latency would be 18 months, from 54 months (6 months before Kindergarten entry) to 6 years (72 months). This paper uses a clear pre-Kindergarten assessment and compares it to early elementary school assessments, thus meeting our criteria.*

- For each study except those using continuous measures, it would be useful to have the information of how many children are considered “at risk” or “below threshold” at the preschool developmental assessment. Considering the public health perspective adopted by the authors, this is an important information. The prevalence of the outcome, when dichotomous, is also necessary to interpret these data.

*Adding additional information (i.e. a further column) would either require removing a different column or further shrinking the font size and it was felt this may influence the readability of an already busy table. However, we have added this instead in a supplementary file (S2 Table) and referred to this in the text (Pg 14 ).*

- Less important: definitions of sensitivity, specificity and predictive values is given, but not the meaning of DOR. Could you consider giving all definitions?

*We have added the definition into pg5.*

- Also less important: the beginning of the Discussion is very similar to the introduction. It is probably possible to jump more quickly to the summarize of the results (although difficult to summarize….)

*We have removed some of the text at the start of the discussion.*
