# Supplementary material for: The predictive value of universal preschool developmental assessment in identifying children with later educational difficulties: A systematic review
Source: PLoS One. 2021 Mar 4;16(3):e0247299. doi: 10.1371/journal.pone.0247299 (PMC7932552; doi:10.1371/journal.pone.0247299)
Supplement: Attachment — Submitted filename: Response to reviewers_22Jan21.docx [file pone.0247299.s007.docx]

**Response to reviewers**

Editor comments: I concur with R1 that the kappa values for agreement at the title and abstract level are low; however, my own experience with systematic reviews has been similar. I would request that you provide more information about the coding procedures: for instance, were *all* titles and *all* abstracts coded by at least two people, or only a subset? When coders disagreed at this level, what processes and/or principles were used to reach a resolution? Being explicit about this process will be particularly important if only a subset of titles and abstracts were coded by more than a single rater. It would also be helpful to provide information about the specific criteria that the raters were using to evaluate titles and abstracts.

*We have added into the study selection section the following text providing further detail about coding procedures:*

*At title and abstract screening two reviewers (LD & RW) independently assessed all papers and in cases of disagreement a resolution was sought between these two reviewers (LD & RW). At full text screening and risk of bias scoring, two reviewers (AK & DC) independently assessed all papers. Where there was disagreement a final decision was made by a third supervising author (RW). Any disagreement at the data extraction stage was discussed between the two reviewers (AK & DC) and resolution sought from a third supervising author (RW). At all points, resolution involved reference to the review protocol to ensure consistency of decision making.*

Reviewer #1: Overall this manuscript has greatly improved and is highly relevant and needed work in this specific area. There are a few minor areas to consider and are outlined below. First, for the study selection the kappa levels appear a bit low and do vary quite a bit based on what was presented. The authors should address why these values vary and why the .31 is considered good, when others would consider this low or weak agreement. How does this impact the results and the study limitations? A justification for the "bespoke checklist" is needed. The authors should discuss how this impacts the study especially in terms of design and rigor. Finally there remains some issues concerning the diagnostic accuracy levels used. The authors state that they have used an 80% diagnostic accuracy rating and have labeled this as a "good" accuracy level. However, work by Plante and Vance (1994) as well as other state that 80% accuracy is fair and 90% is good. I would suggest that the authors do a more complete reading and justification of this decision in their study design.

*We agree with the reviewer that the kappa levels vary substantially, and in some places are fairly low, however, as the editor points out, this is not uncommon in this type of review, and we have been as rigorous as possible in addressing any disagreements. As stated above we have added some further detail on disagreement and coding procedures. We have also adjusted the terms used to more accurately reflect our cited source, although we appreciate others have differing thoughts on the labelling of the different levels.*

*With regards to the diagnostic accuracy labels, we have changed this in the summary measures text to accurately reflect Plante and Vance, and we have checked in the results that this does not alter any results discussed.*

Reviewer #2: Thank you for your additionnal work on this paper, which makes the message clearer.

however, "baby-tests" does non refer to newborn examination, but to developmental scales, developped from Gesell works. Baby-tests include Balyley scales for example.

I understood that you chose not to include them because they cannot be part of universal screening programs. Is it true? I may be worth specify.

*Thank you for the clarification. These sorts of tests, such as the Bayley Scales, potentially could have been included as our criteria state that we were looking for papers involving universal assessment of children as part of whole population child health programmes or population based cohort studies. Within that, we were then broadly agnostic as to the method of development assessment used, but it is inevitably the case that the kinds of studies which were included in these types of studies will tend to involve relatively brief assessment methods that can be applied at scale, rather than more detailed assessments used for review of children at high risk of/with suspected developmental problems. The Bayley Scales offer a fairly detailed assessment of children’s developmental status, and we did not identify any studies meeting our wider inclusion criteria that used that specific approach to developmental assessment.*
